# Preparation of Cardiac Extracts from Embryonal Hearts to Capture RNA–protein Interactions by CLIP

**DOI:** 10.21769/BioProtoc.4857

**Published:** 2023-10-20

**Authors:** Giulia Buonaiuto, Valeria Taliani, Carmine Nicoletti, Fabio Desideri, Monica Ballarino

**Affiliations:** 1 Department of Biology and Biotechnologies “Charles Darwin”, Sapienza University of Rome, Rome, Italy; 2 EMBL, Genome Biology Unit, Heidelberg, Germany; 3 DAHFMO-Unit of Histology and Medical Embryology, Sapienza University of Rome, Rome, Italy; 4 Center for Life Nano- and Neuro-Science, Istituto Italiano di Tecnologia (IIT), Rome, Italy

**Keywords:** Embryonal cardiomyocytes, Cardiomyocyte isolation, RNA, LncRNA, RNA–protein interactions, CLIP

## Abstract

The interaction of RNA with specific RNA-binding proteins (RBP) leads to the establishment of complex regulatory networks through which gene expression is controlled. Careful consideration should be given to the exact environment where a given RNA/RBP interplay occurs, as the functional responses might depend on the type of organism as well as the specific cellular or subcellular contexts. This requisite becomes particularly crucial for the study of long non-coding RNAs (lncRNA), as a consequence of their peculiar tissue-specificity and timely regulated expression. The functional characterization of lncRNAs has traditionally relied on the use of established cell lines that, although useful, are unable to fully recapitulate the complexity of a tissue or organ. Here, we detail an optimized protocol, with comments and tips, to identify the RNA interactome of given RBPs by performing cross-linking immunoprecipitation (CLIP) from mouse embryonal hearts. We tested the efficiency of this protocol on the murine pCharme, a muscle-specific lncRNA interacting with Matrin3 (MATR3) and forming RNA-enriched condensates of biological significance in the nucleus.

Key features

• The protocol refines previous methods of cardiac extracts preparation to use for CLIP assays.

• The protocol allows the quantitative RNA-seq analysis of transcripts interacting with selected proteins.

• Depending on the embryonal stage, a high number of hearts can be required as starting material.

• The steps are adaptable to other tissues and biochemical assays.

**Graphical overview**


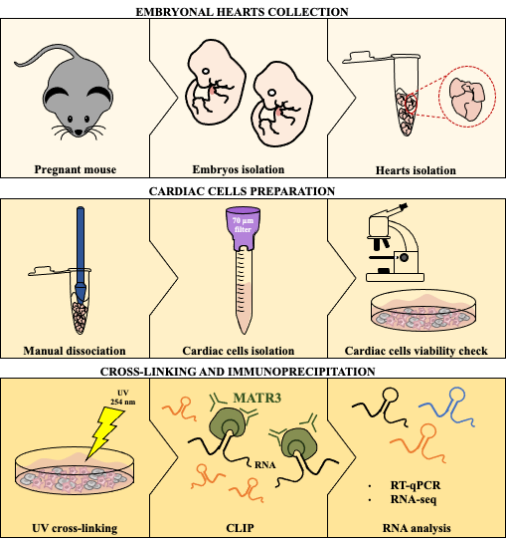


**Identification of RNA/protein interactions from developing hearts**

## Background

The dynamic interaction between RNA and proteins controls many aspects of gene expression and disease [1, 2]. RNA binding proteins (RBP) have been shown to affect every aspect of RNA metabolism by positively or negatively regulating transcription and splicing, cytoplasmic export, and stability of different classes of RNAs [3]. RBP can also regulate mRNA subcellular localization and promote localized translation [4]. On the other hand, non-coding regulatory sequences in the mRNA, such as 5′ or 3′ untranslated regions (UTR), can reciprocally influence protein fate by controlling protein translation [5]. The synergistic interplay between molecular partners often occurs through the formation of dynamic RNA and protein-containing condensates. In the nucleus, this spatial distribution offers many advantages for the processes of RNA transcription and processing since it concentrates the molecular machinery necessary for gene expression in the three-dimensional space. Notably, the importance of RNA/RBP interactions was shown to be favored by noncoding RNAs. Long non-coding RNAs (lncRNAs), in particular, can guide and influence their protein interactome in both nuclear and cytoplasmic compartments due to their structural versatility and highly context-specific expression [6–12]. Over time, many different approaches have been developed to study RNA–protein interactions [13–14]. These methodologies can be classified into two main types: protein-centric or RNA-centric. The first class is based on the possibility to precipitate a specific protein of interest with an antibody to identify its RNA interactome, while the second class uses modified oligos to precipitate an RNA of interest and then characterize the proteins bound to it. However, several examples in the literature highlight the high rate of false positives that characterize these techniques. Indeed, there is a high possibility to observe artefact interactions between RNA and proteins that can occur in vitro after cell lysis [13, 15, 16]. For this reason, one of the essential advancements made in the identification of in vivo RNA/protein interactions is to catch direct binding between molecules while they are occurring in viable cells thanks to the application of different cross-linking methods. In particular, the UV light wavelength of 254 nm is highly efficient for the cross-linking of RNA and proteins in close contact [17] during a cross-linking immunoprecipitation (CLIP) experiment. Another improvement is in the use of fresh tissues as starting substrate to capture the RNA/protein interactions in a more physiological context. However, since the purification of viable cells from tissues can be challenging, few examples of their use are available [18–20].

Here, we present an optimized protocol that, starting from fresh tissue (embryonal murine hearts), allows the preparation of in vivo cardiac extracts suitable for UV cross-linking and subsequent immunoprecipitation assays. The described steps allow the maintenance of in vivo RNA/protein interactions, which is an essential prerogative to identify functional molecular partners involved in the regulation of a biological process.

## Materials and reagents

**Biological materials**

1. C57BL/10 wild-type (WT) mouse (JAX, catalog number: 000665)

**Reagents**

1. Ethanol (Sigma, catalog number: 32221)
2. Dulbecco’s phosphate buffered saline (PBS) (Sigma, catalog number: D8662)
3. Protein inhibitor complex (PIC) (Roche, catalog number: 11873580001)
4. Ribolock (Thermo Fisher Scientific, catalog number: EO0381)
5. Phenylmethylsulfonyl fluoride (PMSF) (Roche, catalog number: 10837091001)
6. Dynabeads (Thermo Fisher Scientific, catalog number: 10004D)
7. Tri reagent (Zymo Research, catalog number: R2050-1-200)
8. HEPES (Sigma, catalog number: H3375)
9. KCl (Sigma, catalog number: P9541)
10. EDTA (Sigma, catalog number: EDS)
11. NaF (Sigma, catalog number: 201154)
12. NP40 (Sigma, catalog number: I3021)
13. Tween 20 (Sigma, catalog number: P9416)
14. Dithiothreitol (DTT) (Roche, catalog number: 10708984001)
15. Tris–HCl (Sigma, catalog number: T1503)
16. NaCl (Sigma, catalog number: S9888)
17. SDS (PanReac Applichem, catalog number: A3942)
18. Proteinase K (Roche, catalog number: 3115828001)
19. Laemmli sample buffer (Bio-Rad, catalog number: 1610747)
20. Anti-MATR3 antibody [Bethyl, catalog number: A300-591A (western 1:500; IP 5 μg)]
21. Anti-IgG antibody (Immunoreagents Inc, catalog number: Rb-003-N; 5 μg)
22. Direct-zol^TM^ RNA MiniPrep (Zymo Research, catalog number: R2050)
23. SuperScript VILO cDNA Synthesis kit (Thermo Fisher Scientific, catalog number: 11754050)
24. PowerUp SYBR-Green MasterMix (Thermo Fisher Scientific, catalog number: A25742)
25. Primers:
  pCharme FW 5′-tttctgtttgccctggacac-3′
  pCharme RV 5′ - gcactcttccttctctccga- 3′
  mCharme FW 5′-ggcacagacaccaaggccag-3′
  mCharme RV 5′ - gcactcttccttctctccga- 3′
  Gapdh FW 5′-tgacgtgccgcctggagaaa-3′
  Gapdh RV 5′-agtgtagcccaagatgcccttcag-3′

**Solutions**

1. PBT (PBS + 0.02% Tween 20)
2. Dissociation media (see Recipes)
3. NP40 lysis buffer (see Recipes)
4. HighSalt NP40 wash buffer (see Recipes)
5. Proteinase K buffer (see Recipes)

**Recipes**

1. **Dissociation media (1 mL)**
*Note: This volume is intended for 10–12 embryonal hearts. Prepare fresh on the day of the experiment.*
  **Table:** 

Reagent	Final concentration	Volume
PBS		0.983 mL
PIC (1 tablet in 500 μL of RNase/DNase-free water)	1×	10 μL
PMSF (0.1 M)	1×	10 μL
Ribolock (40 U/μL)	1:300	3.3 μL
**Total (optional)**		**1 mL**
2. **NP40 lysis buffer (50 mL)**
*Note: Solution can be stored at +4 °C for up to six months. On the day of the experiment, add 0.5 mM DTT (1 M stock), 1× PIC, and 1:200/400 Ribolock to the needed volume (ideal volume of reaction 900 μL for each condition).*
  **Table:** 

Reagent	Final concentration	Volume
HEPES (pH 7.5) (1 M)	50 mM	2.5 mL
KCl (1 M)	150 mM	7.5 mL
EDTA (0.5 M)	2 mM	200 μL
NaF (1 M)	1 mM	50 μL
NP40 (100 %)	0.5%	250 μL
H_2_O		39.5 mL
Total (optional)	n/a	50 mL
3. **HighSalt NP40 wash buffer (50 mL)**
*Note: Solution can be stored at +4 °C for up to six months.*
  **Table:** 

Reagent	Final concentration	Volume
HEPES (pH 7.5) (1 M)	50 mM	2.5 mL
KCl (5 M)	500 mM	5 mL
NP40 (10 %)	0.05%	250 μL
H_2_O		42.250 mL
Total (optional)	n/a	50 mL
4. **Proteinase K buffer (50 mL)**
*Note: Solution can be stored at room temperature for up to six months. On the day of the experiment, add 0.5 mM DTT (1 M stock), 1× PIC, and 1:200/400 Ribolock to the needed volume.*
  **Table:** 

Reagent	Final concentration	Volume
Tris–HCl (pH 7.5) (1 M)	100 mM	5 mL
NaCl (1 M)	150 mM	7.5 mL
EDTA (0.5 M)	12.5 mM	1.25 mL
SDS (20 %)	2%	5 mL
H_2_O		31.25 mL
Total (optional)	n/a	50 mL

**Laboratory supplies**

1. 1.5 mL tube (Sarstedt, catalog number: 72.706)
2. 15 mL tube (Corning, catalog number: 430791)
3. Petri dish 100 mm, tissue culture treated (Corning, catalog number: 353003)

## Equipment

1. Cell strainer 70 μm (Miltenyi Biotec, catalog number: 130-098-462)
2. Cell lifter (Biologix, catalog number: 70-2180)
3. Surgical scissors (F.S.T, catalog number: 14060-10)
4. Jewelers’ forceps, Dumont No. 5, L 4 1/4 (Sigma, catalog number: F6521)
5. Tweezer (Millipore, catalog number: XX6200006P)
6. Micropestle (Geneaid, catalog number: MP050)
7. Microscope (Zeiss, model: Axio Vert.A1)
8. UV-Crosslinker (Spectronics corporation, model: XL-1000)
9. Magnetic rack (Millipore, catalog number: 20-400)
10. Rotating wheel Lab roller (Labnet, catalog number: H5500)
11. Sonicator (Diagenode, model: Bioruptor plus, catalog number: B01020001)
12. Thermomixer (Eppendorf)

## Procedure

This protocol is suitable for the preparation of cellular extract from UV-crosslinked cells isolated from embryonal hearts. Cardiac tissue can be highly heterogeneous and composed of different cell types, such as cardiomyocytes, cardiac fibroblasts, and endothelial cells. Due to their size (lower than strainer cutoff), all these cells are kept during the filtering step, which is mainly required to remove clumps and tissue debris. To maintain in vivo interactions, we recommend the use of freshly collected hearts as cells need to be cross-linked while they are still viable. The cardiac extracts can be used as input for CLIP assay. An IgG negative control should always be run in parallel to the specific IP to check the specificity of the antibody (see sections D–G and [11]). It is important to note that at least 1 mg of total extract per condition (IP-specific and IgG negative control) is necessary. In Taliani et al. (2023) [11], a total of ~60 hearts (E15.5) were collected for CLIP and yielded ~5.3 mg of protein extract (0.09 mg for each E15.5 heart).

- **Embryonal hearts isolation**
  1. Sacrifice the pregnant mouse by CO_2_ or cervical dislocation as approved by the Institutional Animal Use and Care Committee.
  2. Wet the skin and the fur of the mouse with 70% ethanol to avoid samples contamination with mice hair.
  3. Position the mouse under the hood. Pinch the skin with tweezers, pull up and incise and pull apart the skin to expose the abdomen. Cut the peritoneum: you will see all the embryos contained in the placenta (Figure 1A, upper panel).
    **Figure 1.**
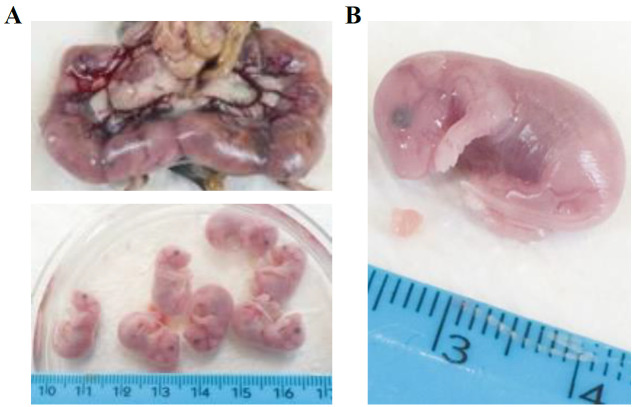
Collection of developing hearts from mouse embryos. A. Representative image of mouse (E15.5) embryos enveloped in the placenta sack. Isolated single embryos are shown below. B. Zoom-in image of representative E15.5 mouse embryo and heart. Ruler is shown for measurement assessment.
  4. Carefully cut the placenta to extract the embryos. Place the embryos in a Petri dish filled with PBS to wash away the blood (Figure 1A, lower panel).
  5. Cut the heads of the embryos and open the chest cavity to collect the hearts (Figure 1B). Depending on the developmental stage (E10.5–E15.5), hearts can be very small (1–3 mm).
  6. Transfer the hearts to a 1.5 mL tube with 1 mL of cold PBS buffer.
*Tip: To facilitate manual dissociation (section B), do not store more than 5–6 hearts in a single 1.5 mL tube.*
- **Manual dissociation**
  ***Attention:*** *Prepare 1 mL of dissociation media. This volume is intended for 10–12 E15.5 embryonal hearts.*
  1. Carefully remove as much PBS as possible to let the hearts settle down to the bottom of the tube (Figure 2A, left).
    **Figure 2.**
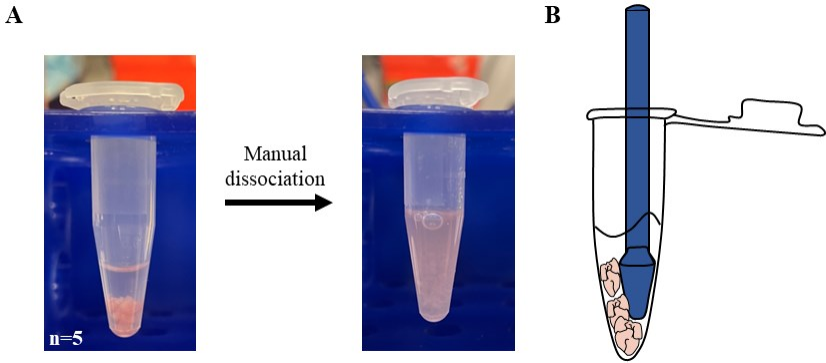
Preparation of cardiac extract. A. 1.5 mL tubes containing E15.5 hearts (n = 5) resuspended in PBS before (left) and after (right) manual dissociation with a pestle. B. Graphic representation of the procedure used to speed up dissociation by trapping the embryonal hearts between pestle and tube.
  2. Add 500 μL of cold dissociation media to each tube (5–6 hearts).
  3. Mash the hearts with a pestle for 2–4 min on ice.
  4. Further dissociate the tissue by pipetting the solution with a 1,000 μL tip until no big clumps of tissue can be observed (Figure 2A, right).
*Tip: For a faster dissociation, trap the hearts between the pestle and the edge of the 1.5 mL tube (Figure 2B). It is possible to follow the dissociation state by looking through the tube against a source of light.*
- **Cardiac cells preparation**
  1. Place the 70 μm strainer (Neonatal Heart Dissociation Kit) on a 15 mL tube on ice.
  2. Slowly add the cardiac homogenates to the strainer with a P1000 pipette. The solution will be filtered with gravity (Video 1).
    **Video 1.**
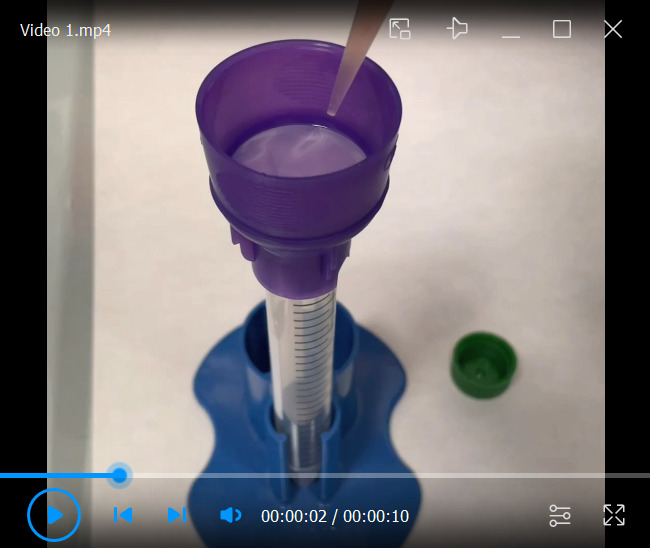
Filtration of cardiac homogenates using a cell strainer
  3. Wash the strainer with PBS until 5 mL of volume is reached in total.
  4. Use a cell lifter to facilitate the filtration process by gently scraping the top of the strainer. Be careful not to break the filter membrane (Video 2).
    **Video 2.**
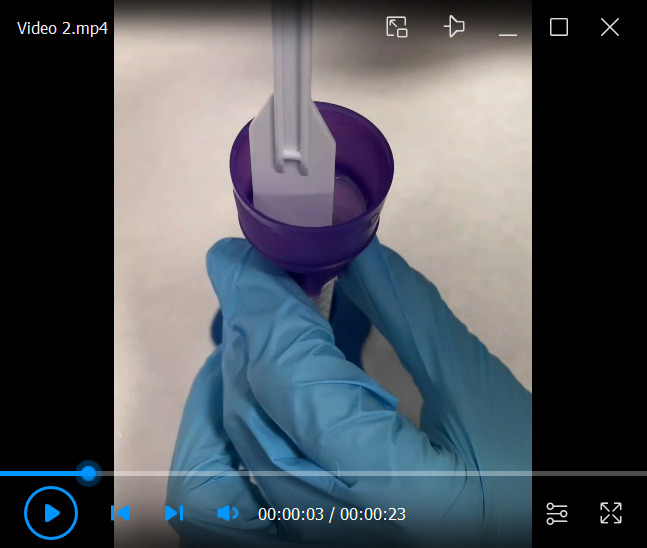
Cell lifter facilitates the filtration process
  5. Pour the 5 mL of filtered cardiac homogenates in one Petri dish (100 mm of diameter) at room temperature and check cells’ viability.
*Tip: Slightly move the plate, and consequently the media, to better visualize the translucent cells under a brightfield light microscope. Cells should stay in suspension (Figure 3). To visualize the cardiac cells, fluorescent or colorimetric dyes can also be used on a small volume of the filtered homogenates.*
    **Figure 3.**
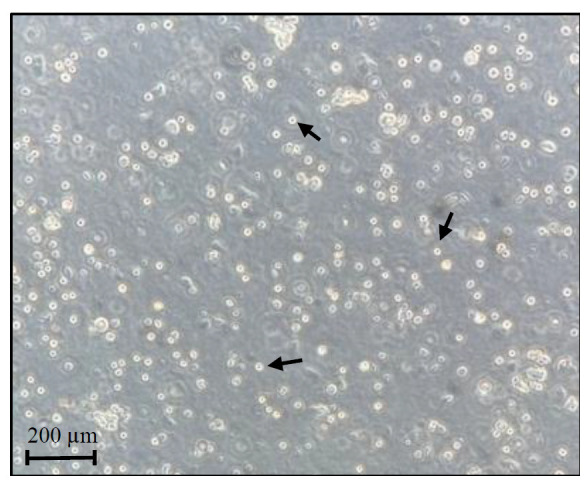
Cardiac cells preparation. Representative image of freshly isolated cells before UV-cross-linking step. Black arrows indicate examples of viable cells.
- **Extract preparation**
  1. Remove the plate lid and UV-crosslink the cells in a Spectrolinker UV Crosslinker at 254 nM with 4,000 μJ/cm^2^ on ice.
  2. Harvest the cells using a cell strainer and transfer the cell suspension to a 15 mL tube.
  3. Centrifuge at 600× *g* for 5 min at 4 °C.
  4. Remove the supernatant very gently to avoid disturbing the cell pellet and snap-freeze the cell pellet on liquid nitrogen. Pellets can be stored at -80 °C for up to 12 months. **Attention:** To proceed with extract preparation, scale up the number of hearts and repeat sections A–B.
  5. Resuspend the frozen pellets in 3 mL of NP40 lysis buffer.
  6. Pipette up and down with a P1000 pipette until the solution becomes homogeneous. Split the volume evenly in three 1.5 mL tubes (1,000 μL each).
  7. Incubate the tubes for 10 min at 4 °C with gentle shaking.
  8. Perform six cycles of sonication at low intensity (see instrument manual) for 30 s at 4 °C with a Bioruptor Plus sonication device to ensure nuclear membrane lysis.
  9. Centrifuge at 16,000× *g* for 5 min at 4 °C and collect the supernatant, which represents the total cellular extract. **Attention:** After centrifugation, the supernatant should be clear. If the sample is still turbid, perform an additional centrifugation (16,000× *g* for 5 min at 4 °C) and repeat steps D5–D9 using a lower quantity of NP40 lysis buffer (1 mL instead of 3 mL).
  10. Quantify the protein extract concentration with Bradford assay. Then, dilute the sample in supplemented NP40 lysis buffer to adjust the final concentration to 1 mg/mL.
- **Beads loading**
  ***Attention:*** *Prepare 1 mL of PBT supplemented with 1× PIC and 1:200/400 Ribolock. This volume is intended for the 2 h incubation of two reactions (IP and IgG). The washing volume is not included.*
  1. For each immunoprecipitation, use two 1.5 mL tubes labeled IP (protein-specific) and IgG (negative control). Gently mix the Dynabeads Protein G magnetic particles and add 30 μL of beads to each tube.
  2. Wash the beads by adding 1 mL of PBT per tube.
  3. Place the tubes on the magnetic rack. Let the beads settle towards the magnetic rack and carefully remove all the supernatant.
  4. Repeat steps E2–E3. Carefully, aspirate the last PBT wash without touching the beads.
  5. Remove tubes from the magnetic rack and resuspend the beads in 400 μL of PBT by pipetting.
  6. Add the same amount of IP-specific or IgG antibodies to the corresponding tubes. Use antibody (Ab) amounts recommended by the manufacturer (usually 5–10 μg of Ab/condition). **Attention:** Use the same Ab isotype for IP and IgG in order to use the same beads in the following section (F).
  7. Incubate at room temperature on a rotating wheel for 2 h.
- **Immunoprecipitation (IP)**
  1. Wash the IP-specific and IgG-loaded beads twice at room temperature with 1 mL of PBT. After each wash, place the tubes on a magnetic rack, let the beads settle, and carefully remove all the supernatant.
  2. Take 10% volume of extract as INPUT (INP) and keep on ice.
  3. On ice, mix 1 mL (1 mg) of the extract with the IP-loaded beads and 1 mL of the extract with the IgG-loaded beads.
  4. Incubate at 4 °C overnight on a rotating wheel.
  5. The day after, wash the beads three times with 1 mL of HighSalt NP40 wash buffer.
  6. Resuspend the beads in 100 μL of NP40 lysis buffer.
  7. Bring the INP sample to 100 μL of final volume with NP40 lysis buffer.
  8. For each sample (IP, IgG, and INP), split the volume in two tubes for RNA and protein extraction. **Attention:** It is possible to split the 100 μL in different combinations. In Taliani et al. (2023) [11], a 3:1 v/v ratio was used for RNA (75 μL) and protein (25 μL) extraction.
- **RNA preparation**
  1. Add 125 μL of proteinase K buffer and 50 μL of proteinase K enzyme to the 75 μL of resuspended beads (RNA tube) to reach a final volume of 250 μL.
  2. Incubate at 50 °C for 30 min with gentle shaking.
  3. Spin the tubes and prepare the sample for RNA extraction by adding 3–5 volumes of Tri reagent.
  4. Let the beads settle toward a magnetic rack and transfer the supernatant to a new tube. Repeat twice. **Attention:** Make sure that no residue of beads is left before proceeding. Bead contaminations can interfere with RNA extraction.
  5. Extract and purify the total RNA to use in gene expression analyses (see section I). In Taliani et al. (2023) [11], the RNA was extracted using the Direct-zol^TM^ RNA MiniPrep kit. Other methods can also be used (e.g., phenol-chloroform extraction). INP usually yields ~1 μg of RNA in a total of 30 μL of RNase/DNase free H_2_O.
- **Protein preparation**
  1. Add 4× Laemmli sample buffer (10 μL) and 50 mM DTT (0.5 M stock, 4 μL) to 25 μL of resuspended beads (protein tube) to reach a final volume of ~40 μL.
  2. Heat at 95 °C for 5 min.
  3. Let the beads settle toward a magnetic rack and transfer the supernatant to a new tube.
  4. Store at -80 °C until western blot analysis (see section I).
- **CLIP experiment analysis**
  1. Check the efficacy of the target protein immunoprecipitation by western blot assay. Load half (20 μL) of the volume of each sample (INP, IP, and IgG) into the SDS-PAGE gel. Proceed with protein separation by gel electrophoresis, transfer, and immunodetection. Western blot analysis for MATR3 is shown in Figure 4 [upper panel, adapted from Taliani et al. (2023) [11], as an example of the expected outcome.
    **Figure 4.**
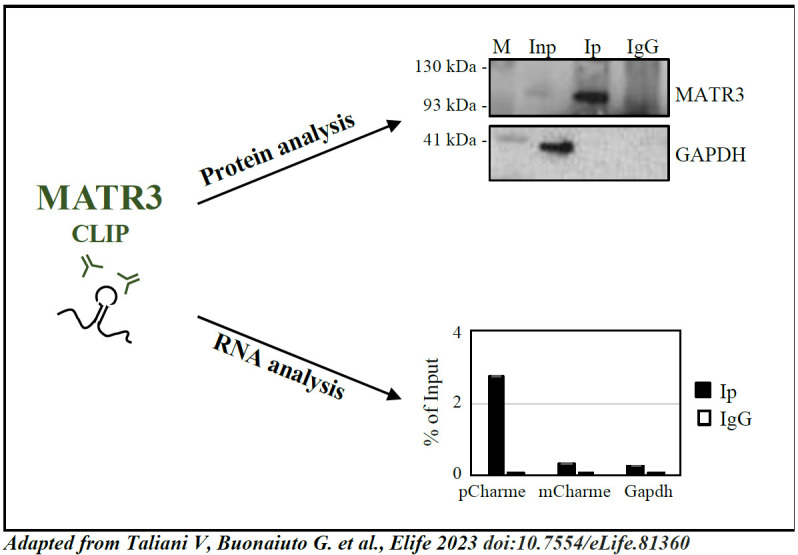
Analysis of CLIP results [adapted from Taliani et al. (2023) [11]. Upper panel, protein analysis: western blot analysis performed on protein samples from MATR3-CLIP assay. GAPDH protein serves as a loading control. INPUT (Inp) samples represent 10% of the total protein extracts. Lower panel, RNA analysis: RT-qPCR performed on RNA samples from MATR3-CLIP assay. pCharme enrichments were compared to mCharme and Gapdh transcripts, both used as negative controls [7, 11] in MATR3 Ip and IgG RNA samples. RT-qPCR quantification is expressed as percentage (%) of Input.
  2. After confirmation of protein recovery, proceed with RNA analysis by RT-qPCR or RNA-sequencing. In Taliani et al. (2023) [11], the RNA samples were retrotranscribed using SuperScript VILO cDNA Synthesis kit and quantified by RT-PCR using PowerUp SYBR-Green MasterMix (hold stage: 2 min at 50 °C, 2 min at 95 °C; amplification: 40× (3 s at 95 °C, 30 s at 60 °C); melt curve: 15 s at 95 °C, 1 min at 60 °C, 30 s at 95 °C, 15 s at 60 °C). RNA was then sequenced on an Illumina Novaseq 6000 Sequencing system.
*Tip: Check the outcome of CLIP by performing RT-qPCR analysis on RNA transcripts used as controls. Figure 4 (lower panel) [adapted from Taliani et al. (2023) [11]] shows an example of RT-qPCR on positive [pCharme, Desideri et al. (2020) [7]] and negative (mCharme and Gapdh) controls for MATR3 CLIP experiment.*

## Data analysis

RNA samples from CLIP assays are suitable for both RT-qPCR and RNA-sequencing. For RT-qPCR, the enrichment of target RNAs can be graphed as the ratio of IP over INP. In the case of RNA sequencing, a detailed description of the analysis is presented in the Material and Methods section of Taliani et al. (2023) [11] (MATR3 CLIP-seq analysis). For the identification of reliable RBP RNA interactors, at least two biological replicates must be processed.

## Validation of protocol

This protocol (or parts of it) was used and validated in the following research article(s):

- Taliani et al. (2023) [11]. The long noncoding RNA Charme supervises cardiomyocyte maturation by controlling cell differentiation programs in the developing heart. Elife (Figure 4, all panels; Figure 4–figure supplement 1, all panels).

## General notes and troubleshooting

**General notes**

1. The cell composition from heart tissue dissociation is not pure but consists of different fractions of cardiomyocytes, cardiac fibroblasts, and endothelial cells. This heterogeneity could cloud the results if your protein/RNA target is lowly expressed or expressed in an underrepresented population of cells.
2. The protocol can be adapted to different starting substrates (e.g., neonatal hearts). Other tissues can also be used depending on the efficacy of viable cell purification.

**Troubleshooting**

Problem 1: The quantity of cells obtained from the hearts is low

Possible cause: Low starting material

Solution: Prepare and freeze multiple cell pellets

Problem 2: The solution does not flow through the 70 μm strainer

Possible cause: The remaining cell clumps in the sample clog the strainer

Solution: Recover the solution from the strainer, use a micropipette to further disrupt cell clumps, and add PBS to dilute the sample. Load the solution in the strainer, keeping the micropipette perpendicular to the strainer membrane, and apply some pressure on the filter without breaking the membrane.
